# Liver contusion in man reveals a Tako-tsubo cardiomyopathy without chest pain: reality or illusion?

**DOI:** 10.11604/pamj.2014.17.176.3461

**Published:** 2014-03-07

**Authors:** Zine el abidine Benali, Hatim Abdedaim, Driss Omari

**Affiliations:** 1 Department of Anesthesiology & Intensive Care, CHP Eddarak, Berkane, Morocco; 2 Department of Anesthesiology & Intensive Care, Military Hospital Mohammed V, University Mohammed V Souissi, Rabat, Morocco; 3 Department of Internal Medicine and Cardiovascular Diseases, CHP Eddarak, Berkane, Morocco

**Keywords:** Liver contusion, Tako-tsubo cardiomyopathy, chest pain, ECG

## Abstract

Tako-tsubo syndrome is very rare in male patients, often overlooked by practitioners in its atypical form painless, and who did not always a good prognostic, often revealed in a context of acute stress at any time in the hospital or outside, its pathophysiology remains to discuss, the diagnosis is greatly facilitated by imaging including echocardiography with apical ballooning. We relate this clinical case of a patient admitted to the ICU for a liver contusion with a diagnosis incidentally this syndrome.

## Introduction

Tako-tsubo (TC) syndrome in the context of closed abdominal trauma and without chest pain is very rarely reported in the medical literature and also presents a difficult differential diagnosis especially with the myocardial infarction and myocardial contusion, referring us through this clinical case of a patient admitted to the ICU for a secondary liver contusion injury of the highway.

## Patient and observation

**Figure 1:**
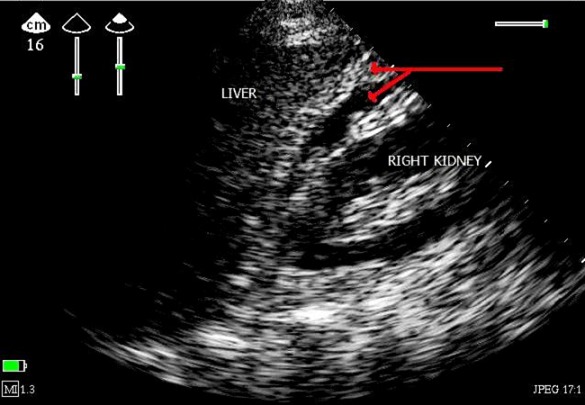
Abdominal ultrasonography mode 2 D showed a effusion at the Morrison's pouch with low abundance and contusion of the lower edge of the liver (red arrow)V

**Figure 2:**
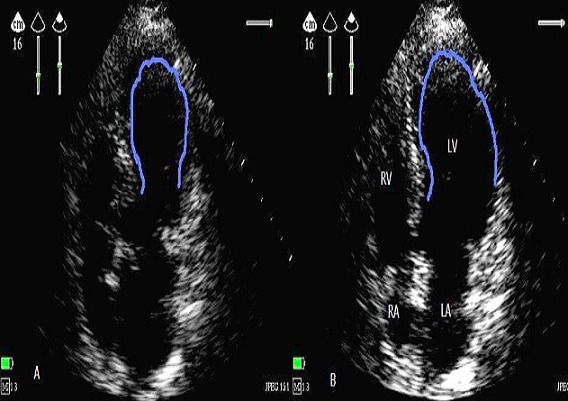
Echocardiogram on admission demonstrated apical akinesis with hyper kinesis basal and ballooning of the left ventricle apex (blue arc); A End-systolic phase, B End-diastolic phase.(LA: left atrium, LV: left ventricle, RV: right ventricle, RA: right atrium)

**Figure 3:**
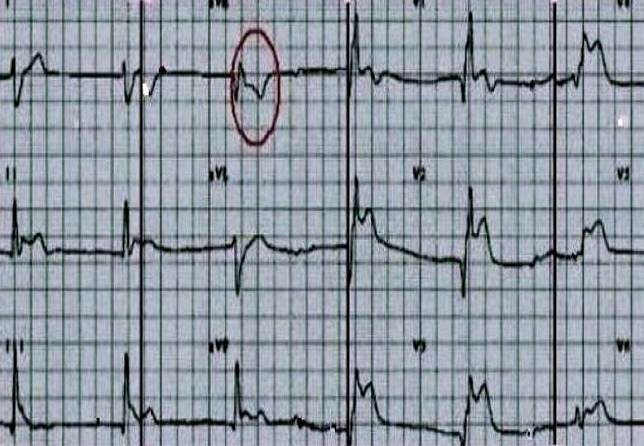
Electrocardiogram demonstrated ST segment elevation in lead DIII V2 V3 V4 V5 V 6 and specially -aVR (red ellipse)

Referring us to a 60 year old male patient, without pathological antecedents, accident victim of the highway with the point of abdominal impact, admitted to the ICU for monitoring, review the admission showed a well oriented in space and time, personality anxious, the palpebral conjunctivae were not pale, stable blood pressure with 120/70 mm hg, painful on palpation of the abdomen in the right upper quadrant, the examination of the chest is normal without pain, cardiac examination is completely normal except for a slight bradycardia to 54 beat / min. ultrasound abdominal (Figure 1) showed a perihepatic effusion of low abundance with contusion of the lower edge of the liver, echocardiography (Figure 2) bedside was systematically whatever reason in any patient admitted to the ICU, showed in our patient: a bollonisation with akinesia ( lack of mobility and thickening myocardial) apical with hyper kinesis basal, the fraction ejection estimated at 45%, no valvulopathy associated, dry pericardium, the ascending and abdominal aorta without abnormality, size and respiratory compliance of the inferior vena cava were normal. This prompted us to do an electrocardiogram outside any chest pain showed that: ST segment elevation in DIII V2 V3 V4 V5 V6 and - aVR (Figure 3), the troponin Ic showed a moderate increase of 2 µg/L, the blood electrolytes and blood count without any special, before the clinical and biological aspect asked cardiac MRI outpatient near hospital (because of the unavailability of technical trays for coronary angiography) showed that left ventricular ballooning with myocardial edema without necrosis or fibrosis and therefore diagnosis of TC syndrome was retained. The patient was put on analgesic, diuretic and platelet aggregation inhibitors without anticoagulants as because of the risk of worsening liver bleeding in a traumatic context. A second echocardiogram after one week showed a total resolution of apical akinesia formally illustrating the TC syndrome.

## Discussion

The TC syndrome (literally a trap octopus is an instrument several days left on the seabed in Japan to fish), also called apical ballooning syndrome is a cardiomyopathy consisting of myocardial stunning occurring after emotional stress. This affection was first described by Japanese cardiologists and internists in the 80 [1]. The most recent published study in 2013 about TC syndrome (analysed in multicentre registry) shows that Among 179 consecutive patients with proven diagnosis of TS, a women represented the majority of patients 94% while men 6% developed TS. Mean age was 69.1±11.5 years (range 35–88 years) and the chest pain is present in 82% [2]. It is probably caused by intense and sudden discharge of catecholamines the waning of intense stress. The mechanism of impaired cardiac function is not clear. However it could be a reversible disorder coronary microcirculation, not visible by coronary angiography. In addition, the concentration of beta-adrenergic receptor is greater at the tip of the ventricle, which could contribute to a differential response in the parts of the heart after adrenergic stimulation [3]. In our patient, the stress situation is probably triggered by abdominal trauma associated with anxious personality. The clinical presentation is similar to acute Myocardial infarction (MI), with chest pain, dyspnea, and ECG changes. A diagnosis of TC cardiomyopathy is always challenging and should be routinely considered in the differential diagnosis of acute MI. Proposed Mayo Clinic criteria (widely but not universally accepted) for TC cardiomyopathy include: Transient hypokinesis, akinesis, or dyskinesis of the LV mid segments, with or without apical involvement; regional wall motion abnormalities extending beyond a single epicardial vascular distribution. A stressful trigger (often but not always present). The absence of obstructive coronary disease or angiographic evidence of acute plaque rupture. New ECG abnormalities (either ST segment elevation and/or T wave inversion) or modest elevation in cardiac troponin. The absence of pheochromocytoma or myocarditis [4]. Our patient had signs electrical, biological including Moderate increase in troponin and echocardiographic but without chest pain knowing that′s not diabetic, he presented only the right upper quadrant pain related to the trauma. Another differential diagnosis is required: it is the traumatic myocardial contusion (MC). Although the precise diagnosis of MC remains a matter of controversy, an accurate assessment of the heart is required after trauma. Even if very low possibility of increased intrathoracic pressure transmitted by a compression of the abdomen (as in our case report) can enter the MC, this diagnosis is not retained because the lesions most often affect the right ventricle and the septum [5]. In addition, the myocardial necrosis appears less homogeneous and confined to specific muscular branches [6], and does not give a typical left ventricular apical ballooning as in the TC syndrome.

Recently, Kosuge et al demonstrated that diffuse ST segment elevation is associated with extensive distribution of wall-motion abnormalities centered on the apex. All these ST segment shifts extended beyond the perfusion territory of any single coronary artery, and not explain the impairment of only one coronary artery. This might serve as one of the key points in differentiating TC syndrome from acute myocardial infarction. In TC syndrome, ST segment elevation most frequently occurred in lead -aVR, because this Lead faces the apical and inferolateral regions, which none of the standard 12 leads face directly,Where the importance of analyzing aVR and - aVR [7]. TC syndrome had a much lower prevalence of ST segment elevation in lead V1 compared to Anterior Wall ST Segment elevation Acute Myocardial Infarction and ST segment elevation in ≥1 of leads V_3_ to V_5_ without ST segment elevation in lead V_1_ identified TC syndrome with sensitivity of 74.2% and specificity of 80.6% [8]. Our patient presented these electric signs.

Echocardiography will reveal marked left ventricular dysfunction. More specifically, the wall motion abnormalities extend beyond the distribution of any single coronary artery and include moderate to severe dysfunction the apical segment of the left ventricle with sparing and usually hyperkinesis of the basal segment [9] However, the definitive diagnosis of stress cardiomyopathy is confirmed when echocardiography repeated after few days to weeks shows complete normalization of regional wall motion abnormalities and left ventricular ejection fraction. Our patient had typical sonographic signs of apical ballooning with a total resolution of akinesia in 7 days, It is the systematic use of echocardiography bedside whatever the reason for hospitalization in the ICU who helped us to put the diagnosis of TC syndrome without chest event. Generally in the imaging MRI In all cases of TC syndrome, the most characteristic finding is ventricular edema that appears as high signal intensity with a transmural distribution and the location of the edema is not related to a vascular territory of coronary arteries, edema is distributed in both the apical and mid planes of the LV, also the absence of perfusion defects and delayed enhancement are other clues that differentiate TC syndrome from other diseases,The perfusion sequence in patients with TC syndrome usually is normal. These features can be used to differentiate TC syndrome from acute myocardial infarction, in which edema usually has a transmural effect on the LV wall but always has a vascular distribution. The presence of apical akinesis produces the ballooning morphology of the LV and hyperkinesis in the basal plane that characterizes Tako-tsubo cardiomyopathy [10].

Overall, In hospital cardiovascular complications occurred about 14% and consisted principally of cardiac arrhythmias, cardiogenic shock, cardiac decompensation and cardiovascular death, evolution, though generally smooth, can be marked by a rupture of the left ventricle that makes this cause a recently identified of sudden death in the hospital or outside [2, 11]. Which requires early diagnosis and adequate monitoring, our patient has not presented these complications.

## Conclusion

Without discussion the coronarography is the gold standard in the diagnosis of tako-tsubo syndrome but without forgetting the values that bring us: notably echocardiographic analysis by practitioners trained in echocardiography, electrocardiogram, troponin and MRI, help us a lot to ask this diagnosis often unnoticed especially without apparent chest pain, which is sometimes revealed by dramatic complications including sudden unexplained death in a situation of stress for the other pathology.
